# FLT3-ITD confers resistance to the PI3K/Akt pathway inhibitors by protecting the mTOR/4EBP1/Mcl-1 pathway through STAT5 activation in acute myeloid leukemia

**DOI:** 10.18632/oncotarget.3279

**Published:** 2015-03-16

**Authors:** Ayako Nogami, Gaku Oshikawa, Keigo Okada, Shusaku Fukutake, Yoshihiro Umezawa, Toshikage Nagao, Tetsuya Kurosu, Osamu Miura

**Affiliations:** ^1^ Department of Hematology, Graduate School of Medical and Dental Sciences, Tokyo Medical and Dental University, Tokyo, Japan

**Keywords:** AML, FLT3, PI3K, STAT5, MCL-1

## Abstract

FLT3-ITD and FLT3-TKD are the most frequent tyrosine kinase mutations in acute myeloid leukemia (AML), with the former associated with poor prognosis. Here, we show that the PI3K inhibitor GDC-0941 or the Akt inhibitor MK-2206 induced apoptosis through the mitochondria-mediated intrinsic pathway more efficiently in hematopoietic 32D cells driven by FLT3-TKD (32D/TKD) than FLT3-ITD (32D/ITD), which robustly activated STAT5. The resistance to GDC-0941 and MK-2206 was gained by expression of the constitutively activated STAT5 mutant STAT5A1*6 in 32D/TKD cells, while it was abrogated by the STAT5 inhibitor pimozide in 32D/ITD cells or FLT3-ITD-expressing human leukemic MV4–11 cells. GDC-0941 or MK-2206 induced dephosphorylation of 4EBP1 more conspicuously in 32D/TKD than in 32D/ITD, which was prevented or augmented by STAT5A1*6 or pimozide, respectively, and correlated with downregulation of the eIF4E/eIF4G complex formation and Mcl-1 expression. Furthermore, exogenous expression of Mcl-1 endowed resistance to GDC-0941 and MK-2206 on 32D/TKD cells. Finally, it was confirmed in primary AML cells with FLT3-ITD that pimozide enhanced 4EBP1 dephosphorylation and Mcl-1 downregulation to augment cytotoxicity of GDC-0941. These data suggest that the robust STAT5 activation by FLT3-ITD protects cells treated with the PI3K/Akt pathway inhibitors from apoptosis by maintaining Mcl-1 expression through the mTORC1/4EBP1/eIF4E pathway.

## INTRODUCTION

FMS-like tyrosine kinase 3 (FLT3) is a receptor-tyrosine kinase with a split tyrosine kinase domain (TKD), TKD1 and TKD2, expressed on hematopoietic progenitors and regulates early steps of hematopoietic progenitor cell proliferation, survival, and differentiation [1, 2]. In acute myeloid leukemia (AML), FLT3 is widely expressed and is mutated in about one third of patients. Internal tandem duplication (ITD) mutations in the juxtamembrane domain of FLT3 (FLT3-ITDs) are the most frequent kinase mutation in AML, occurring in 25–30% of cases. Point mutations within TKD2 (FLT3-TKDs), such as the most predominant D835Y mutation, are found in 5–10% of patients with AML. While the clinical significance of FLT3-TKD in AML has still remained controversial, it has been well established that FLT3-ITD confers a poor prognosis mostly due to higher relapse rates leading to inferior disease-free and overall survivals, which is particularly significant in AML with higher mutant to wild-type allelic ratio [3, 4]. Moreover, recent studies have revealed that the insertion site of ITD in TKD1, observed in about one quarter of the cases, as well as a high ITD allelic ratio is associated with aggressive clinical features with higher peripheral blast count and intrinsic therapy resistance with lower complete response rates [5–8]. Both types of mutations cause ligand-independent activation of the FLT3 receptor, leading to constitutive activation of the various downstream signaling pathways that are normally activated by ligand-stimulated FLT3, such as the PI3K/Akt and MEK/ERK pathways, thus leading to cytokine-independent survival and proliferation of model hematopoietic cell lines and myeloproliferative disorders in various murine models [1, 2]. Importantly, FLT3-ITD but not FLT3-TKD strongly activates STAT5 in various cell lines as well as in primary murine and human FLT3-ITD positive leukemic cells, which may contribute to enhanced transforming potentials of FLT3-ITD as compared with FLT3-TKD in cellular and murine models [9–12].

It has been well established that the PI3K/Akt signaling pathway plays an important role in both normal and malignant hematopoiesis [13, 14]. This pathway represents a promising target for leukemia therapy, because it is frequently activated in various leukemias, including FLT3-mutated AML, and thought to correlate with poor prognosis and drug resistance. As a consequence, a large number of pharmacological inhibitors against PI3K and Akt have been developed and under preclinical or clinical studies as well as in clinical usage. Of these inhibitors, GDC-0941 (pictilisib) and MK-2206 are oral, potent and selective inhibitors for the class I PI3Ks and all Akt isoforms, respectively, showing promising effects in preclinical studies and have been well-tolerated in phase I clinical studies [15, 16]. However, the effects of these inhibitors on AML cells have been only modest [14]. Therefore, further studies are warranted to elucidate the mechanisms underlying the resistance of AML cells to the PI3K/Akt pathway inhibitors and to develop new therapeutic strategies to circumvent this resistance.

The mTOR signaling pathway is mainly activated downstream of the PI3K/Akt pathway in a variety of circumstances and plays key roles in regulation of survival, growth, and metabolism of a variety of cells [13, 17, 18]. Of the two multi-protein complexes formed by the serine/threonine kinase mTOR, mTORC1 and mTORC2, the former plays a critical role in regulation of cap-dependent translation of mRNAs through phosphorylation of 4EBP1. The phosphorylation leads to the release of 4EBP1 from the mRNA m^7^-GTP cap-binding protein eIF4E to allow its interaction with the scaffolding protein eIF4G to initiate the formation of the translation-initiating complex eIF4F required for the translation of mRNAs containing long 5′-UTRs, which are highly structured and have a high G+C content. A previous study has shown that FTL3-ITD activates the mTORC1 signaling pathway to promote survival of FLT3-ITD-expressing AML cells [19]. One of the most important downstream targets of the mTOR/4EBP1/eIF4E pathway is the anti-apoptotic Bcl-2 family member Mcl-1, which is highly unstable and, to maintain its expression level, requires active cap-dependent translation of its mRNA with a G+C rich 5′ UTR [20–22]. Mcl-1 plays a crucial role in survival of hematopoietic progenitor cells and various malignant hematopoietic cells including AML cells [23, 24]. It has been reported that FLT3-ITD as well as ligand-stimulated FLT3 increases the expression of Mcl-1, which promotes cell survival and may confer resistance to chemotherapeutic agents on these cells [25, 26]. However, possible roles of the mTOR/4EBP1/eIF4E pathway in upregulation of Mcl-1 and regulation of apoptosis in FLT3-ITD-expressing cells have remained to be determined.

In the present study, we demonstrate that FLT3-ITD as compared with FLT3-TKD confers resistance to the PI3K/Akt pathway inhibitors through the robust activation of STAT5, which may partially protect the 4EBP1 phosphorylation to maintain the eIF4F formation and Mcl-1 expression. The present study may contribute to the development of novel therapeutic strategies against intractable AML with FLT3-ITD, because inhibition of STAT5 was found to synergistically enhance cytotoxic effects of the PI3K/Akt pathway inhibitors by downregulating Mcl-1 expression in AML cells expressing FLT3-ITD.

## RESULTS

### Inhibition of the PI3K/Akt pathway induces apoptosis through the intrinsic pathway more prominently in 32D cells transformed by FLT3-TKD than by FLT3-ITD

**Figure 1:**
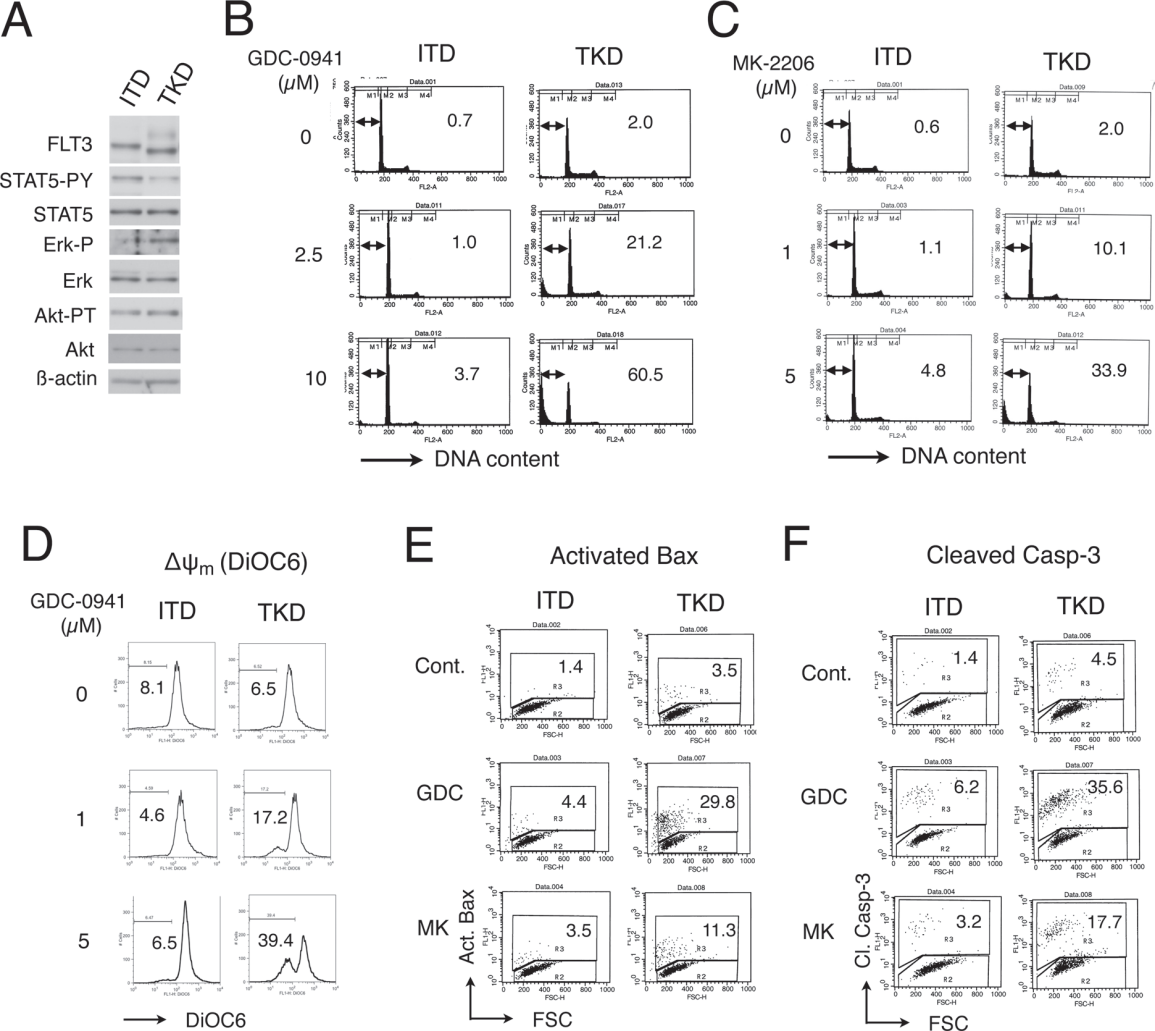
GDC-0941 and MK-2206 induces apoptosis through the intrinsic pathway more prominently in 32D cells transformed by FLT3-TKD than by FLT3-ITD **A.** 32D/ITD (ITD) or 32D/TKD (TKD) cells were lysed and subjected to Western blot analysis with antibodies against indicated proteins. Abbreviations used are: STAT5-PY, phospho-Y694-STAT5; Erk-P, phospho-T202/Y204-Erk; Akt-PT, phospho-T308-Akt. (B, C) 32D/ITD (ITD) or 32D/TKD (TKD) cells were cultured with indicated concentrations of GDC-0941 **B.** or MK-2206 **C.** for 42 h and analyzed for the cellular DNA content by flow cytometry. Percentages of apoptotic cells with sub-G1 DNA content are indicated. **D.** 32D/ITD (ITD) or 32D/TKD (TKD) cells were cultured with indicated concentrations of GDC-0941 for 24 h and analyzed for the mitochondrial membrane potential (Δψ_m_) by flow cytometry as described in Materials and Methods. Percentages of cells with reduced Δψ_m_ are indicated. (E, F) 32D/ITD (ITD) or 32D/TKD (TKD) cells were left untreated as control (Cont.) or treated with 5 μM of GDC-0941 or MK-2206 for 24 h and analyzed for the activated Bax (Act. Bax) **E.** and cleaved Caspase-3 (Cl. Casp-3) **F.** by flow cytometry. Percentages of cells with activated Bax or Caspase-3 are indicated.

To address the possibility that cells expressing FLT3-ITD and FLT3-TKD may show different sensitivities to inhibition of the PI3K/Akt pathway, we analyzed 32D/ITD and 32D/TKD cells, which are murine hematopoietic cells transformed by FLT3-ITD and FLT3-TKD as described in Materials and Methods. As shown in Fig. 1A, we first confirmed that STAT5 or Erk was activated more significantly in FLT3-ITD or FLT3-TKD cells, respectively, while Akt was activated similarly in these cells, in accordance with previous reports [11, 12, 27]. Next, we examined effects of GDC-0941 (pictilisib), a selective inhibitor targeting all class I PI3K isoforms, on induction of apoptosis in these cells. As shown in Fig. 1B, GDC-0941 induced apoptosis remarkably in 32D/TKD cells in a dose-dependent way as judged by increases in cells with sub-G1 cellular DNA content, which is a hallmark for apoptotic cells. On the other hand, apoptosis was only faintly induced in 32D/ITD cells by GDC-0941 at concentrations up to 10 μM. Furthermore, the Akt inhibitor MK-2206 also induced apoptosis much more prominently in 32D/TKD cells than in 32D/ITD cells (Fig. 1C). In accordance with these data, GDC-0941 and MK-2206 more significantly inhibited proliferation and reduced viability of 32D/ITD cells than 32D/TKD cells as demonstrated by growth curves and dose-effect analyses of cell proliferation (Supplemental Fig. S1A, S2). Next, we examined the mechanisms involved in induction of apoptosis in these cells and found that GDC-0941 as well as MK-2206 induced decline in mitochondrial membrane potential (Δψ_m_), activation of Bax, and cleavage of Caspase-3 much more prominently in 32D/TKD cells than in 32D/ITD cells (Fig. 1D–F). These results indicate that 32D/ITD cells are relatively resistant to the PI3K/Akt pathway inhibitors, which prominently induced apoptosis in 32D/TKD cells through the mitochondria-mediated intrinsic pathway involving activation of Bax and Caspases.

### The STAT5 inhibitor pimozide abrogates resistance against GDC-0941 and MK-2206 in 32D/ITD cells and in MV4–11 leukemic cells expressing FLT3-ITD

**Figure 2:**
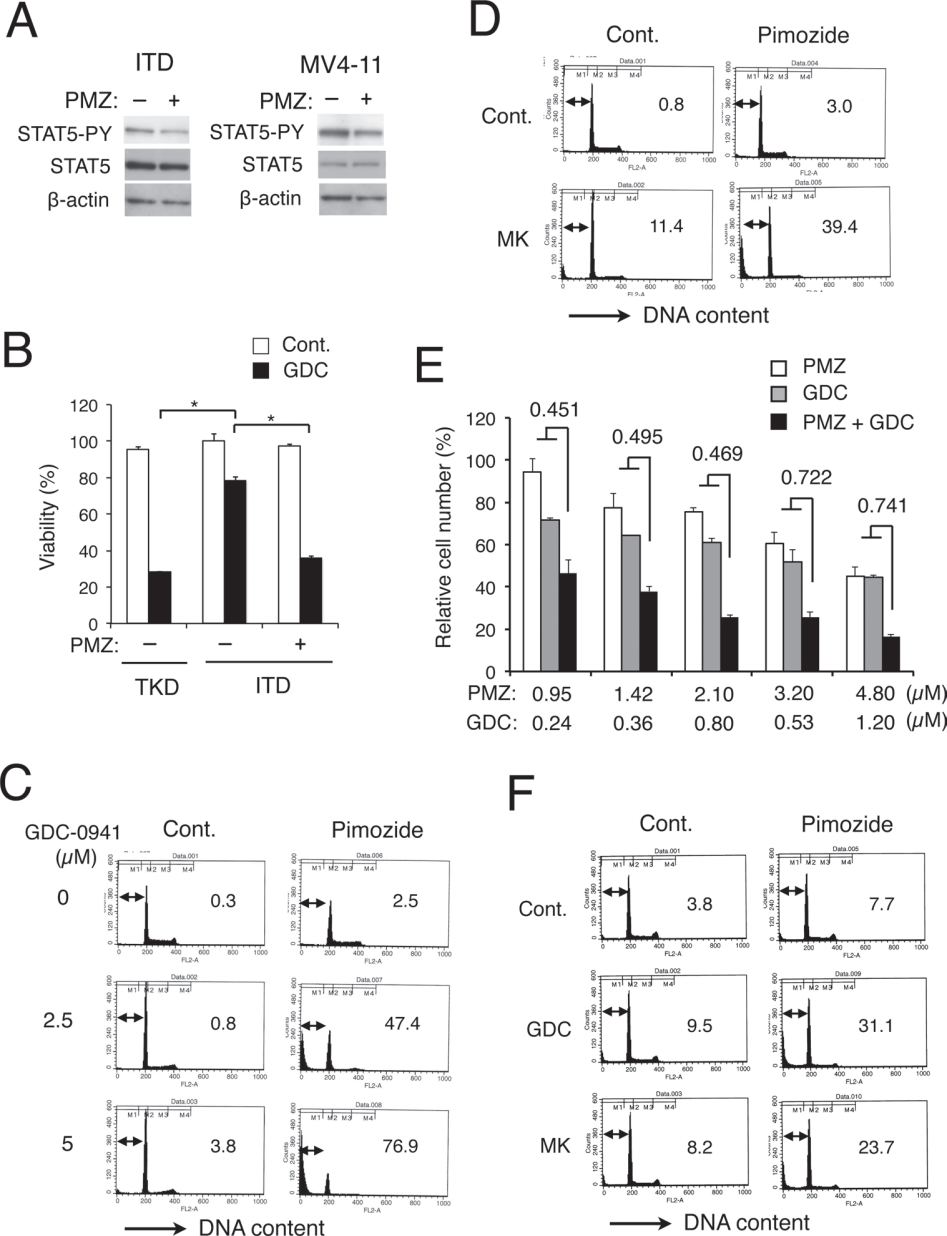
The STAT5 inhibitor pimozide abrogates resistance against GDC-0941 and MK-2206 in FLT3-ITD cells and in MV4–11 leukemic cells expressing FLT3-ITD **A.** 32D/ITD (ITD) or MV4–11 cells were treated with 5 μM pimozide for 1 h or 10 μM pimozide for 4 h, respectively, and subjected to Western blot analyses with antibodies against indicated proteins. Abbreviations used are: PMZ, pimozide; STAT5-PY, phospho-Y694-STAT5. **B.** 32D/ITD (ITD) or 32D/TKD (TKD) cells were left untreated as control or treated with 5 μM GDC-0941 (GDC) with or without 2.5 μM pimozide (PMZ) for 48 h, and cell viability was measured after trypan blue staining. Each column represents the mean of triplicate measurements, with error bars indicating standard errors. The asterisks indicate statistically significant differences determined by Student's *t*-test (*p* < 0.001). **C.** 32D/ITD cells were treated with indicated concentrations of GDC-0941 with or without 5 μM pimozide, as indicated, for 48 h and analyzed for the cellular DNA content by flow cytometry. Percentages of apoptotic cells with sub-G1 DNA content are indicated. **D.** 32D/ITD cells were left untreated as control (Cont.) or treated with 5 μM MK-2206 in the presence or absence of 2.5 μM pimozide, as indicated, for 48 h and analyzed. **E.** MV4–11 cells were cultured with indicated concentrations of GDC-0941 (GDC) and pimozide (PMZ) for 72 h. Viable cell numbers were measured by the XTT colorimetric assay. Each column represents the mean of triplicate determinations, with error bars indicating standard errors, and is expressed as a percentage of cell numbers without inhibitors. Combination index (CI) values obtained by the method of Chou and Talalay [30] are indicated. **F.** MV4–11 cells were left untreated as control (Cont.) or treated with 1 μM GDC-0941 or 3 μM MK-2206 in the presence or absence of 3 μM pimozide, as indicated, for 72 h and analyzed.

Because FLT3-ITD activates STAT5 more prominently than FLT3-TKD, it was speculated that the robust STAT5 activation may be associated with the resistance to GDC-0941 and MK-2206 in 32D/ITD cells. To pursuit this possibility, we examined effects of the STAT5 inhibitor pimozide [28] on cells expressing FLT3-ITD. As shown in Fig. 2A, pimozide reduced the activation-specific phosphorylation of STAT5 on Y694 in 32D/ITD cells as well as in human leukemic MV4–11 cells expressing FLT3-ITD, in accordance with a previous report [29]. As shown in Fig. 2B, it was confirmed that GDC-0941 much less significantly reduced viability of 32D/ITD cells than that of 32D/TKD cells (Fig. 2B). However, pimozide abated the resistance of 32D/ITD cells to GDC-0941 and remarkably augmented its cytotoxic effect, whereas pimozide by itself barely reduced the viability of these cells (Fig. 2B, Supplemental Fig.S1A). In accordance with these data, pimozide synergistically enhanced apoptosis induced by GDC-0941 or MK-2206 in 32D/ITD cells (Fig. 2C and 2D). We next examined the effects of GDC-0941 and pimozide on human AML cells expressing FLT3-ITD, MV4–11. As shown in Fig. 2E, GDC-0941 and pimozide synergistically reduced viable cell numbers of MV4–11, as judged by combination index (CI) values obtained by the method of Chuo and Talalay [30] being less than 1 at all the concentrations examined. In accordance with this, viability of MV4–11 was significantly reduced by the combined treatment of GDC-0941 and pimozide, while these inhibitors alone did not affect its viability (Supplemental Fig. S1B). It was also confirmed that pimozide significantly enhanced apoptosis induced by GDC-0941 or MK-2206 in MV4–11 cells (Fig. 2F). These data suggest that the robust STAT5 activation by FLT3-ITD may play a role in acquisition of the resistance to the PI3K/Akt pathway inhibitors by leukemic cells.

### A constitutively activated STAT5 mutant, STAT5A1*6, confers resistance to GDC-0941 and MK-2206 on 32D/TKD cells

**Figure 3:**
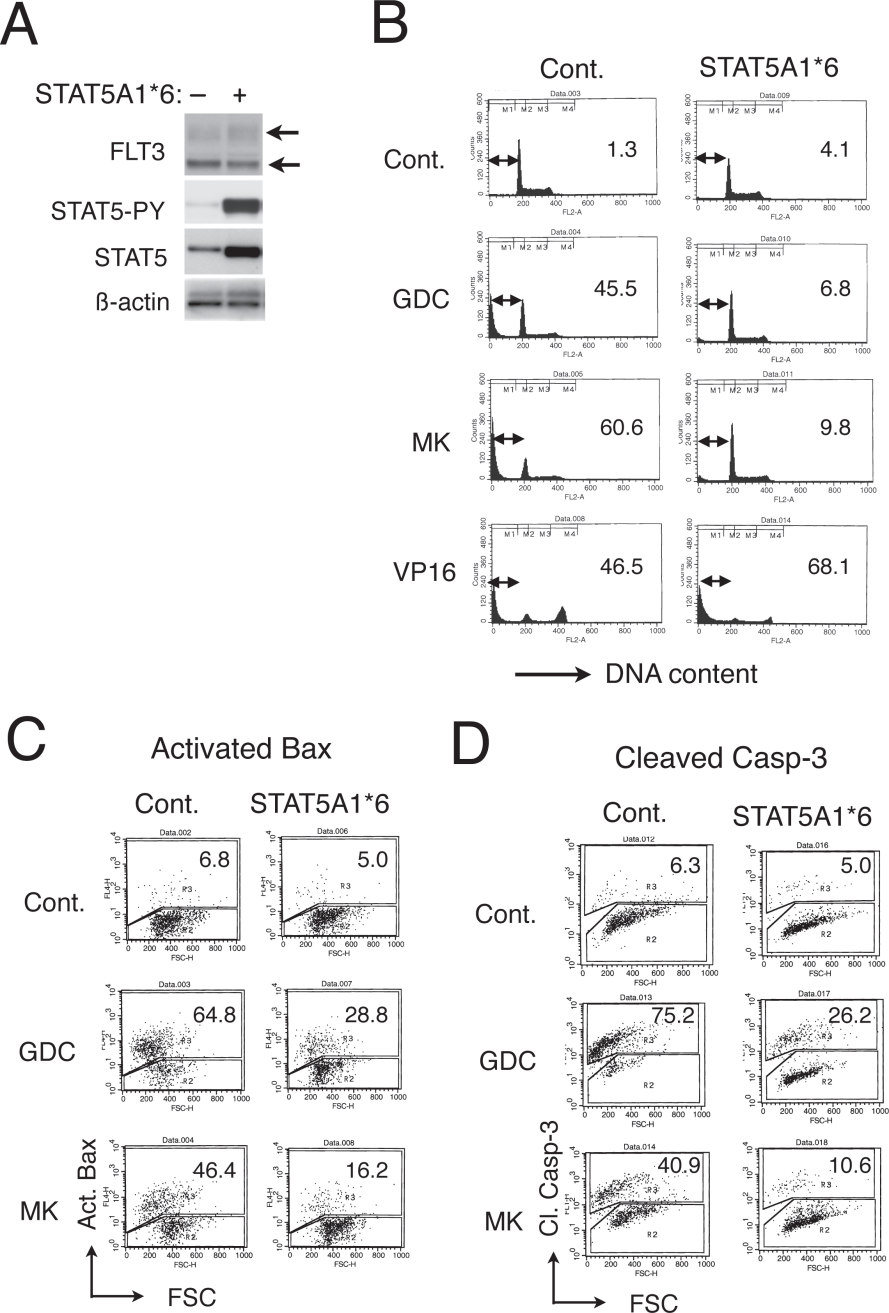
A constitutively activated STAT5 mutant, STAT5A1*6, confers resistance to GDC-0941 and MK-2206 on 32D/TKD cells **A.** 32D/TKD cells transduced with STAT5A1*6 or vector control cells, as indicated, were lysed and subjected to Western blot analyses with antibodies against indicated proteins. Positions of FLT3-TKD are indicated. STAT5-PY, phospho-Y694-STAT5. **B.** 32D/TKD cells transduced with STAT5A1*6 or vector control cells were left untreated as control (Cont.) or treated with 5 μM GDC-0941 (GDC), 5 μM MK-2206 (MK) or 0.5 μM etoposide (VP16), as indicated for 48 h and analyzed for the cellular DNA content by flow cytometry. Percentages of apoptotic cells with sub-G1 DNA content are indicated. (C, D) 32D/TKD cells transduced with STAT5A1*6 or vector control cells were left untreated as control or treated with 5 μM of GDC-0941 or MK-2206 for 24 h, as indicated, and analyzed for the activated Bax (Act. Bax) **C.** or cleaved Caspase-3 (Cl. Casp-3) **D.** by flow cytometry. Percentages of cells with activated Bax or Caspase-3 are indicated.

To confirm the involvement of STAT5 activation in acquisition of the resistance to the PI3K/Akt pathway inhibitors, we expressed a constitutively activated STAT5 mutant, STAT5A1*6, in 32D/TKD cells and examined the effects of GDC-0941 or MK-2206 on these cells. First, it was confirmed that these cells expressed Y694-phosphorylated STAT5 as well as STAT5 at a remarkably higher level as compared with the vector-control cells, while FLT3-TKD was expressed at comparable levels in these cells (Fig. 3A). As shown in Fig. 3B, apoptosis induced by GDC-0941 or MK-2206 was significantly inhibited by expression of STAT5A1*6, whereas that induced by a chemotherapeutic agent, etoposide, was rather enhanced. In accordance with this, GDC-0941 less significantly inhibited proliferation or reduced viability of STAT5A1*6-expressiong 32D/TKD cells than vector control cells (Supplemental Fig. S1C). It was further revealed that STAT5A1*6 unequivocally inhibited activation of Bax and cleavage of Caspase-3 in these cells treated with GDC-0941 or MK-2206 (Fig. 3C, 3D). Together, these data further support the idea that the robust activation of STAT5 by FLT3-ITD may confer resistance to the PI3K/Akt pathway inhibitors by preventing activation of the intrinsic apoptotic pathway.

### FLT3-ITD confers resistance to the PI3K/Akt pathway inhibitors through STAT5 activation to sustain 4EBP1 phosphorylation and Mcl-1 expression to prevent Caspase-9 activation

**Figure 4:**
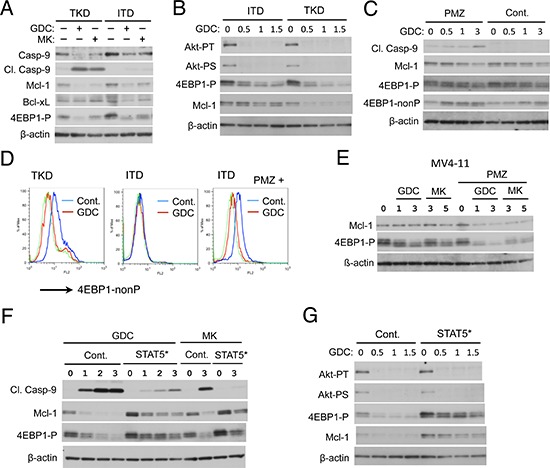
FLT3-ITD confers resistance to the PI3K/Akt pathway inhibitors through STAT5 activation by sustaining 4EBP1 phosphorylation and Mcl-1 expression to prevent Caspase-9 activation **A.** 32D/ITD (ITD) or 32D/TKD (TKD) cells were left untreated as control or treated with GDC-0941 (GDC) or MK-2206 (MK) at 3 μM for 30 h as indicated. Cells were lysed and subjected to Western blot analysis with antibodies against indicated proteins. Abbreviations used are: Casp-9, Caspase-9; Cl. Casp-9; Cleaved Caspase-9, 4EBP1-P, phospho-T37/49–4EBP1. **B.** 32D/ITD (ITD) or 32D/TKD (TKD) cells were treated with indicated concentrations (μM) of GDC-0941 for 4 h and subjected to Western blot analysis. Akt-PT, phospho-T308-Akt; Akt-PS, phospho-S473-Akt. **C.** 32D/ITD cells were treated with indicated concentrations (μM) of GDC-0941 in the presence or absence of 5 μM pimozide (PMZ), as indicated, for 24 h and analyzed. 4EBP1-nonP, non-phospho-T46–4EBP1. **D.** 32D/ITD (ITD) or 32D/TKD (TKD) cells were left untreated as control or treated with 1 μM GDC-0941 in the absence or presence of 5 μM pimozide (PMZ), as indicated, for 4 h. Cells were subjected to flow cytometric analysis with an antibody specific for unphosphorylated form of 4EBP1 (4EBP1-nonP). Cells treated with or without GDC-0941 are shown in a blue or red line, respectively, while a green line represents isotype control for the antibody used. **E.** MV4–11 cells were treated with indicated concentrations (μM) of GDC-0941 or MK-2206 in the presence or absence of 5 μM pimozide (PMZ), as indicated, for 20 h and analyzed. **F.** 32D/TKD cells transduced with STAT5A1*6 (STAT5*) or vector control cells (Cont.), as indicated, were treated with indicated concentrations (μM) of GDC-0941 (GDC) or MK-2206 (MK) for 20 h and analyzed. **G.** 32D/TKD cells transduced with STAT5A1*6 (STAT5*) or vector control cells (Cont.), as indicated, were treated with indicated concentrations (μM) of GDC-0941 for 4 h and analyzed.

To explore the molecular mechanisms by which FLT3-ITD confers resistance to the PI3K/Akt pathway inhibitors, we examined the effects of GDC-0941 or MK-2206 on various factors involved in regulation of apoptosis and proliferation in these cells by Western blot analyses. Fig. 4A shows that Caspase-9 was cleaved specifically in 32D/TKD cells but not in 32D/ITD cells after treatment with GDC-0941 or MK-2206 for 30 h. Thus, under these conditions, the PI3K/Akt pathway inhibitors activated mitochondria-mediated apoptotic pathway selectively in 32D/TKD cells, as Caspase-9 is cleaved and activated just downstream of the mitochondria in this pathway [31]. Because activation of this pathway is protected by anti-apoptotic members of the Bcl-2 family proteins, we examined the expression level of Mcl-1, a critical member of this family in hematopoietic cells, including AML cells [23, 24]. As shown in Fig. 4A, the expression level of Mcl-1 was slightly lower and reduced more conspicuously by GDC-0941 or MK-2206 in 32D/TKD cells than in 32D/ITD cells. On the other hand, the expression level of Bcl-xL was not significantly reduced by these inhibitors in 32D/TKD cells. Because the expression level of Mcl-1 is regulated through translational as well as transcriptional and post-translational mechanisms [31], we examined 4EBP1, which is phosphorylated by mTORC1 downstream of the PI3K/Akt pathway and is known to play a critical role in cap-dependent translational of *Mcl-1* mRNA [20, 21]. As shown in Fig. 4A, phosphorylation of 4EBP1 was reduced by GDC-0941 and MK-2206 to lower levels in 32D/TKD cells as compared with 32D/ITD cells, which correlated with the expression levels of Mcl-1 in these cells. To confirm and extend these observations, we treated these cells with increasing concentrations of GDC-0941 for a shorter period of time (4 h) and examined its effects on Mcl-1 and 4EBP1, because Mcl-1 has a short half-life and may be cleaved by activated caspases in cells undergoing apoptosis. As shown in Fig. 4B, GDC-0941 very efficiently inhibited the activation specific phosphorylation of Akt on T308 comparably in both 32D/ITD and 32D/TKD cells. However, the dose-dependent decline in Mcl-1 expression as well in 4EBP1 phosphorylation was more prominent in 32D/TKD cells than in 32D/ITD cells. Similar results were obtained with MK-2206 (data not shown). These results suggest the possibility that FLT3-ITD may maintain the 4EBP1/Mcl-1 axis downstream of the PI3K/Akt pathway to protect cells from activation of the mitochondrial apoptotic pathway leading to activation of Caspase-9 in these cells.

To investigate the possible role of STAT5 activation in the protective mechanisms involving 4EBP1 and Mcl-1, we next examined the effect of STAT5 inhibitor pimozide in 32D/ITD cells. As shown in Fig. 4C, treatment of 32D/ITD cells for 24 h with GDC-0941 induced the cleavage of Caspase-9 only in the presence of pimozide, which correlated with the decline in Mcl-1 expression. Under these conditions, Western blot analysis with anti-phospho-4EBP1 revealed mainly an increase in electrophoretic mobility of 4EBP1 induced by pimozide in 32D/ITD cells treated with GDC-0941, which implicates enhancement of 4EBP1 dephosphorylation by pimozide. This was confirmed by analysis using an anti-4EBP1 antibody specifically reactive with the unphosphorylated form (Fig. 4C). Flow cytometric analyses using this antibody further confirmed that GDC-0941 treatment for 4 h conspicuously increased the expression level of non-phosphorylated 4EBP1 in the absence of pimozide in 32D/TKD cells but only in the presence of pimozide in 32D/ITD cells (Fig. 4D). In accordance with a previous report [32], neither GDC-0941 nor MK-2206 significantly reduced Mcl-1 expression in MV4–11 cells (Fig. 4E). As expected, however, pimozide synergistically enhanced the decline in Mcl-1 expression and 4EBP1 phosphorylation induced by GDC-0941 or MK-2206.

We next examined the effects STAT5A1*6 expressed in 32D/TKD cells. As shown in Fig. 4F, the expression level of Mcl-1 as well as phosphorylated 4EBP1 was found increased in cells expressing STAT5A1*6. Furthermore, STAT5A1*6 at least partly prevented the decline in 4EBP1 phosphorylation and Mcl-1 expression as well as cleavage of Caspase-9 in 32D/TKD cells treated with GDC-0941 or MK-2206 (Fig. 4F). These effects of STAT5A1*6 on 4EBP1 and Mcl-1 was confirmed in cells treated with GDC-0941 or MK-2206 for 4 h without any significant effect on inhibition of Akt induced by these inhibitors observed (Fig. 4G and data not shown). These results suggest that the robust activation of STAT5 by FLT3-ITD may play a critical role in maintaining the 4EBP1/Mcl-1 pathway downstream of Akt to protect cells treated with the PI3K/Akt pathway inhibitors from apoptosis.

### FLT3-ITD may sustain cap-dependent translation of Mcl-1 through STAT5 activation to prevent apoptosis in cells treated with the PI3K/Akt pathway inhibitors

**Figure 5:**
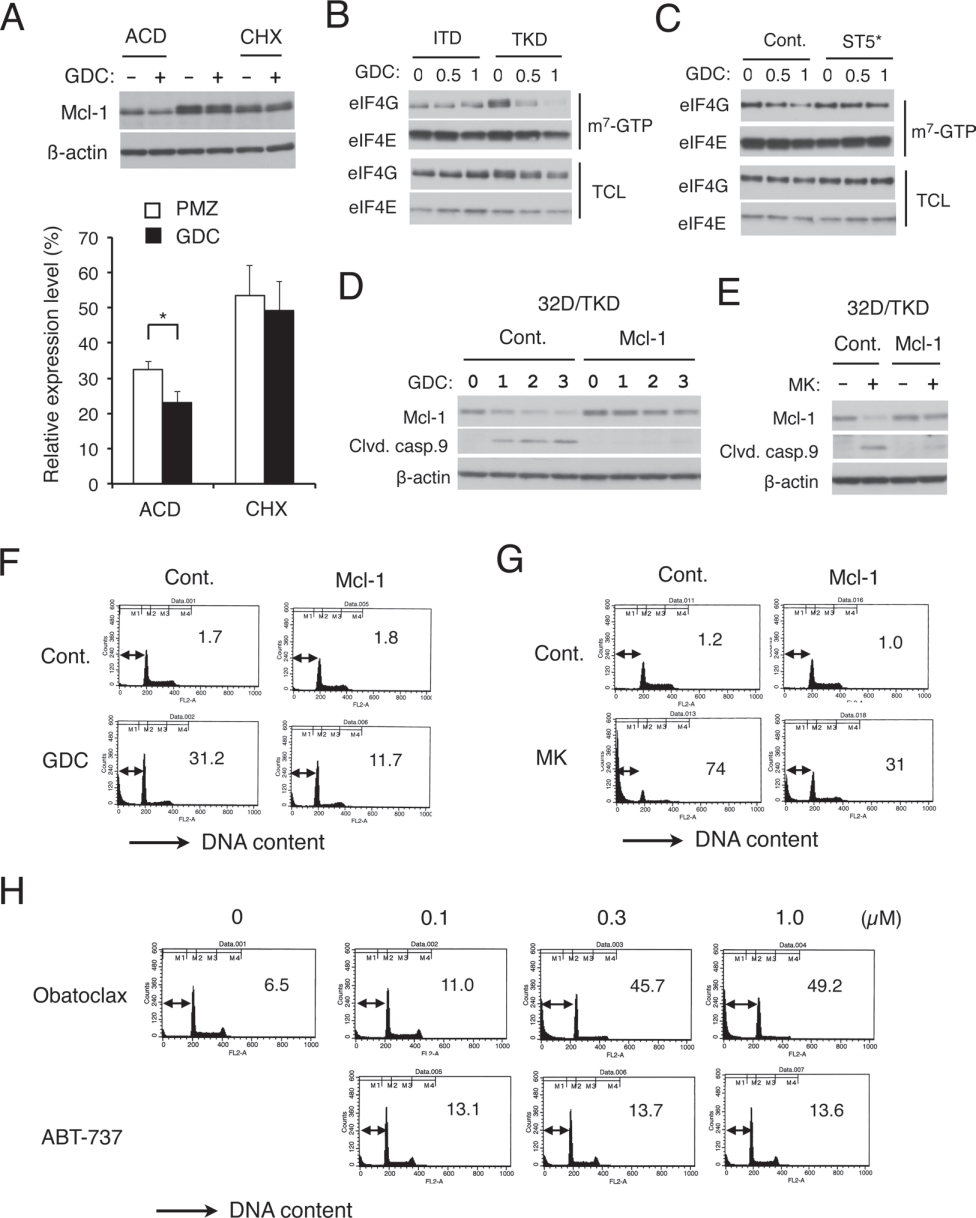
FLT3-ITD may sustain cap-dependent translation of Mcl-1 through STAT5 activation to prevent apoptosis in cells treated with the PI3K/Akt pathway inhibitors **A.** 32D/TKD (TKD) cells were cultured for 1 h with 1 μM GDC-0941 and lysed or cultured further with 5 μg/ml actinomycin D (ACD) for 4 h or with 50 μg/ml cycloheximide (CHX) for 1 h, as indicated, before cell lysis. Cell lysates were subjected to Western blot analysis. Expression levels of Mcl-1 were analyzed by densitometry and expressed as compared with that in cells before treatment with actinomycin D or cycloheximide. Each column represents the mean of 5 independent experiments, with error bars indicating standard errors. The asterisk indicates a statistically significant difference determined by Student's *t*-test (*p* = 0.017). **B.** 32D/ITD (ITD) or 32D/TKD (TKD) cells were treated with indicated concentrations (μM) of GDC-0941 (GDC) for 6 h and subjected to the cap-binding assay to analyze eIF4E-eIF4G complex formation. Proteins bound to m^7^-GTP-sepharose (m^7^-GTP) as well as total cell lysates (TCL) were analyzed by immunoblotting with indicated antibodies. **C.** 32D/TKD cells transduced with STAT5A1*6 (ST5*) or vector control cells (Cont.), as indicated, were analyzed as described for B. **D, E.** 32D/TKD cells transduced with Mcl-1 or vector control cells, as indicated, were treated with indicated concentrations of GDC-0941 (GDC) or 3 μM MK-2206 (MK) for 20 h, as indicated, and subjected to Western blot analysis. **F, G.** 32D/TKD cells transduced with Mcl-1 or vector control cells, as indicated, were left untreated as control (Cont.) or treated with 5 μM of GDC-0941 (GDC) or MK-2206 (MK), as indicated, for 48 h and analyzed for the cellular DNA content by flow cytometry. Percentages of apoptotic cells with sub-G1 DNA content are indicated. **H.** 32D/ITD cells were treated with indicated concentration of obatoclax or ABT-737 for 48 h and analyzed for the cellular DNA content.

We then examined the mechanisms involved in GDC-0941-induced reduction in Mcl-1 expression level, which is known to be regulated through not only translational but also transcriptional and post-translational mechanisms [31]. As shown in Fig. 5A, GDC-0941 significantly facilitated the decline in Mcl-1 expression in 32D/TKD cells treated with actinomycin D to arrest transcription. On the other hand, GDC-0941 failed to accelerate the decline in Mcl-1 expression significantly when translation was arrested by treatment with cycloheximide. These results suggest that Mcl-1 expression may be downregulated by GDC-0941 not mainly through transcriptional or post-translational mechanisms but through translational mechanisms. Thus, we next performed the pull-down assays using m^7^-GTP beads to evaluate the effect of GDC-0941 on formation of the eIF4E/eIF4G complex, which enhances the cap-dependent translation of mRNAs having lengthy, G+C-rich, highly structured 5′ UTRs, such as *Mcl-1* mRNA [20]. As shown in Fig. 5B, GDC-0941 significantly reduced the amount of eIF4G pulled down with eIF4E bound to m^7^-GTP in 32D/TKD cells but not in 32D/ITD cells. Furthermore, expression of STAT5A1*6 in 32D/TKD cells mostly prevented the GDC-0941-induced reduction in eIF4G binding to m^7^-GTP-bound eIF4E (Fig. 5B). These data imply that 4EBP1 dephosphorylated by GDC-0941 treatment in 32D/TKD cells may inhibit formation of the eIF4E/eIF4G complex required for translation of cap-dependent mRNAs, including that for Mcl-1, through mechanisms preventable by the robust activation of STAT5, thus supporting the idea that inhibition of the PI3K/Akt pathway may downregulate Mcl-1 expression in 32D/TKD cells mostly through translational mechanisms involving dephosphorylation of 4EBP1.

To examine the significance of Mcl-1 downregulation in induction of apoptosis induced by the PI3K/Akt pathway inhibitors in 32D/TKD cells, we transduced an expression vector for Mcl-1 into these cells and examined the effects of GDC-0941 and MK-2206. As shown in Fig. 5D and 5E, Mcl-1 was expressed at a moderately higher level in these cells than in vector control cells. It was also confirmed that GDC-0941 or MK-2206 more conspicuously reduced Mcl-1 expression in vector control cells than in cells transduced with the Mcl-1 expression vector, which transcribes the artificial *Mcl-1* mRNA lacking the lengthy, G+C-rich, highly structured 5′ UTRs to be translated efficiently in a cap-independent manner. It was further demonstrated that the exogenous expression of Mcl-1 resistant to downregulation by the PI3K/Akt pathway inhibitors conferred resistance for apoptosis induced by them, as measured by cleavage of caspase-9 and cellular DNA (Fig. 5D–5G). To confirm the significance of Mcl-1 in regulation of cell survival further, we examined effects of the BH3 mimetics ABT-737 and obatoclax on FLT3/ITD cells comparatively. As shown in Fig. 5H, ABT-737 only modestly induced apoptosis in FLT3/ITD cells even at concentrations up to 1 μM. On the other hand, obatoclax induced apoptosis remarkably in these cells in a dose-dependent manner. Because Mcl-1 is inhibited only by the extended-spectrum BH3 mimetic obatoclax, while Bcl-xL as well as Bcl-2 is inhibited by both of these BH3 mimetics, Mcl-1 should play a crucial role in regulation of survival of these cells. Together, these results support the idea that the maintenance of Mcl-1 level by FLT3-ITD in cells treated with the PI3K/Akt pathway inhibitors may play a critical role in protection of cells from apoptosis.

### Pimozide and GDC-0941 synergistically downregulate 4EBP1 phosphorylation and Mcl-1 expression to reduce viability of primary AML cells expressing FLT3-ITD

**Figure 6:**
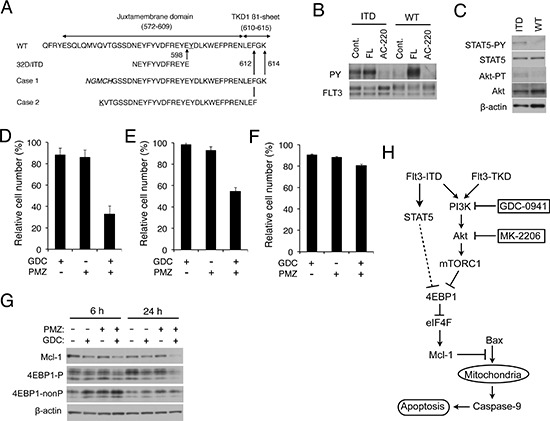
Pimozide and GDC-0941 synergistically enhance downregulation of 4EBP1 phosphorylation and Mcl-1 expression to reduce viability of primary AML cells expressing FLT3-ITD **A.** Partial amino acid sequences of wild-type FLT3 (WT) and inserted amino acid sequences of FLT3-ITD expressed in 32D/ITD or in primary AML cells from Case 1 and 2 are shown. The insertion sites are indicated by arrows and amino-acid numbers. Sequences unrelated to FLT3 found inserted in Case 1 or mutated in Case 2 are italicized or underlined, respectively. **B.** Primary AML cells from Case 1 with FLT3-ITD (ITD) or from a control AML patient with the wild-type FLT3 (WT) were left untreated as control (Cont.) or treated with 10 ng/ml FLT3-ligand (FL) for 15 min or 10 nM AC-220 for 2 h, as indicated, and lysed. Lysates were subjected to immunoprecipitation with anti-FLT3 and analyzed by immunoblot analysis with antibodies against phosphotyrosine (PY) and FLT3, as indicated. **C.** Total cell lysates of primary AML cells from Case 1 (ITD) or from the control AML patient (WT) were analyzed by immunoblotting with indicated antibodies. **D.** Primary AML cells with FLT3-ITD from Case 1 were cultured for 48 h with or without 0.5 μM GDC-0941 (GDC) and 2 μM pimozide (PMZ), as indicated. Numbers of viable cells were measured after trypan blue staining, normalized by that of cells cultured without inhibitors, and plotted. Each column represents the mean of triplicate measurements, with error bars indicating standard errors. **E.** Primary AML cells with FLT3-ITD from Case 2 were cultured for 24 h with or without 0.5 μM GDC-0941 (GDC) and 2 μM pimozide (PMZ), as indicated, and analyzed. **F.** Primary AML cells from the control AML patient with the wild-type FLT3 were cultured for 24 h with or without 0.5 μM GDC-0941 (GDC) and 2 μM pimozide (PMZ), as indicated, in the presence of 10 ng/ml FLT3-ligand and analyzed. **G.** Primary AML cells with FLT3-ITD from Case 1 were cultured for 6 h or 24 h with or without 0.5 μM GDC-0941 (GDC) and 2 μM pimozide (PMZ), as indicated. Cells were lysed and subjected to Western blot analysis with antibodies against indicated proteins. **H.** A schematic model of intracellular signaling mechanisms downstream of FLT3-ITD and FLT3-TKD regulating survival and proliferation of AML cells in response to the PI3K/Akt pathway inhibitors.

We finally examined the effects of GDC-0941 on the 4EBP1/Mcl-1 pathway and survival in primary AML cells expressing FLT3-ITD. For this purpose, we first examined AML cells expressing FLT3 with a long ITD of 96 nucleotides and additional 15-nucleotides coding for 37 amino acids inserted in TKD1 (Case 1 in Fig. 6A). The allelic ratio of this mutation was estimated as approximately 1.0 by PCR and sequencing analyses (data not shown). As shown in Fig. 6B, FLT3 expressed in these cells from Case 1 was constitutively phosphorylated on tyrosine, which was only faintly enhanced by stimulation with FLT3 ligand and was abrogated by the FLT3 inhibitor AC-220. On the other hand, tyrosine phosphorylation of FLT3 could not be detected in control primary AML cells without the ITD mutation and was very strongly induced by stimulation with FLT3 ligand. It was also confirmed that STAT5 was constitutively activated in the primary AML cells with FLT3-ITD from Case 1 but not in the control AML cells (Fig. 6C). It was also demonstrated that Akt was activated more distinctively in the FLT3-ITD cells than in the control AML cells. These data imply that most, if not all, of the primary AML cells with the FLT3-ITD mutation expressed constitutively-activated FLT3-ITD.

We then examined the effects of GDC-0941 and pimozide on viability of the primary AML cells. As shown in Fig. 6D, at concentrations only slightly effective when used individually, combined treatment with GCD-0941 and pimozide remarkably reduced the viable cell number of the primary AML cells from Case 1 expressing FLT3-ITD. Similar results were obtained using different primary AML cells from Case 2 expressing FLT3 with a long ITD of 33 amino acids inserted in TKD1 with the allelic ratio of approximately 1.0 (Fig. 6A, 6E, and data not shown). On the other hand, combined treatment with GCD-0941 and pimozide only modestly enhanced a reduction in viability of control AML cells with the wild-type FLT3 cultured with FLT3 ligand, as compared with when the inhibitors were used alone (Fig. 6F). Furthermore, it was demonstrated that pimozide clearly enhanced the reduction in 4EBP1 phosphorylation and Mcl-1 expression induced by GDC-0941 in AML cells with FLT3-ITD from Case 1 (Fig. 6G). These results suggest that inhibition of PI3K and STAT5 by GDC-0941 and pimozide, respectively, synergistically suppressed the 4EBP1/Mcl-1 pathway leading to reduction in viability of primary AML cells expressing FLT3-ITD.

## DISCUSSION

In the present study, we have revealed that 32D cells transformed by FLT3-ITD as compared with FLT3-TKD are more resistant to the PI3K inhibitor GDC-0941 or the Akt inhibitor MK-2206 for inhibition of proliferation and induction of apoptosis mediated through the mitochondria-mediated intrinsic pathway involving activation of Bax and Caspases (Fig. 1, Supplemental Fig. S1, S2). The robust activation of STAT5 by FLT3-ITD was found to play a role in acquisition of the resistance, because it was reduced by the STAT5 inhibitor pimozide in 32D/ITD cells, MV4–11 cells, or primary AML cells with FLT3-ITD and was acquired through expression of the constitutively activated SATAT5 mutant STAT5A1*6 in 32D/TKD cells (Figs. 2, 3, 6, Supplemental Fig. S1). The resistance to the PI3K/Akt pathway inhibitors as well as the STAT5 activation levels correlated with the levels of 4EBP1 phosphorylation, eIF4E/eIF4G complex formation, and Mcl-1 expression, while the exogenous expression of Mcl-1 conferred the resistance on 32D/TKD cells (Figs. 4, 5, 6). Based on these results, we put forward a schematic model (Fig. 6H), in which the robust activation of STAT5 by FLT3-ITD sustains the Mcl-1 expression through the 4EBP1/eIF4F pathway to alleviate mitochondria-mediated apoptosis in cells treated with the PI3K/Akt pathway inhibitors. Thus, simultaneous targeting of STAT5 and the PI3K/Akt pathway should provide a promising therapeutic strategy for FLT3-ITD-positive AML with poor prognosis.

Mcl-1 is the most critical member of the anti-apoptotic Bcl-2 family playing a crucial role in survival of hematopoietic progenitor cells as well as AML cells including those with FLT3-ITD [23–26]. Consistent with this, downregulation of Mcl-1 expression closely correlated with induction of apoptosis under various conditions in the present study (Fig. 4). Furthermore, overexpression of exogenous Mcl-1, which was resistant to downregulation by GDC-0941 and MK-2206, conferred resistance to apoptosis induced by these inhibitors in 32D/TKD cells (Fig. 5D–G). On the other hand, these inhibitors did not significantly reduce the expression level of Bcl-xL in FLT3-TKD cells under the conditions where reduction in that of Mcl-1 as well as induction of apoptosis was conspicuously observed (Fig. 4A). Moreover, apoptosis was only faintly induced by ABT-737, which inhibits Bcl-xL and Bcl-2 but not Mcl-1, but remarkably induced by obatoclax, which additionally inhibits Mcl-1, in 32D/ITD cells [33, 34] (Fig. 5H). Although Bcl-xL is also expressed at high levels in hematopoietic cells and implicated as a downstream target of STAT5 [35], these data indicate that Mcl-1, but not Bcl-xL, should play a critical role downstream of STAT5 in regulation of apoptosis to confer the relative resistance to the PI3K and Akt inhibitors on cells expressing FLT3-ITD.

Due to the short half-life of *Mcl-1* mRNA and protein, its expression level is modulated rapidly through transcriptional, translational, and post-translational mechanisms in response to various stimuli [31]. For instance, hematopoietic cytokines have been reported to upregulate the *Mcl-1* transcription through STAT5 as well as through the PI3K/Akt pathway [36]. In accordance with this, Yoshimoto G et al. previously reported that knockdown of STAT5 downregulated both mRNA and protein levels of Mcl-1 in MV4–11 cells [25]. Thus, the transcriptional activation of Mcl-1 by the robust activation of STAT5 may explain a moderately higher level of Mcl-1 observed in 32D/ITD or STAT5A1*6-expressing 32D/TKD cells than in 32D/TKD or vector-control cells, respectively (Fig. 4A, B, F, G). On the other hand, because the Akt phosphorylation was inhibited similarly and efficiently in these cells by GDC-0941 or MK-2206 (Fig. 4B, G), the transcriptional downregulation through inhibition of the PI3K/Akt pathway may not explain the difference in downregulation of Mcl-1 (Fig. 4B, G). Finally, the effect of GDC-0941 to decrease the expression level of Mcl-1 was still observed in 32D/TKD cells treated with actinomycin D to shut down transcription (Fig. 5C), thus indicating that the downregulation was at least partly through post-transcriptional mechanisms in these cells.

It is also well established that Mcl-1 expression is regulated post-translationally through cleavage by activated Caspases in apoptotic cells as well as through the ubiquitin/proteasomal degradation pathway [31]. In this regard, it should be noted that the decline in Mcl-1 expression in our study was observed as early as 4 h after treatment with GDC-0941 or MK-2206 and before the activation-specific cleavage of caspases became detectable (Fig. 4B, G and data not shown). The PI3K/Akt pathway and its downstream target GSK3 are known to play roles in downregulation of Mcl-1 through the ubiquitin/proteasome pathway [31]. However, Mcl-1 expression was not significantly affected by GDC-0941 in cells treated with cycloheximide to shut down translation (Fig. 5C), thus indicating that the post-translational mechanisms may not play a significant role in Mcl-1 downregulation induced by the PI3K inhibitor. This idea is strongly supported by the observation that Mcl-1 exogenously expressed in 32D/TKD cells was not significantly affected by GDC-0941 or MK-2206 in contrast to endogenous Mcl-1 (Fig. 5D), thus ruling out the possibility that destabilization of Mcl-1 as the significant mechanism for its downregulation in these cells.

The translation of *Mcl-1* mRNA, with a lengthy G+C-rich 5′ UTR, is mainly regulated by formation of the eIF4F complex [20, 21], which plays a critical role in cap-dependent translation and is directly competed by hypophosphorylated 4EBP1 induced by suppression of the mTOR pathway [18, 22]. In the present study, we observed the close correlation between dephosphorylation of 4EBP1 and downregulation of Mcl-1 induced by the PI3K and Akt inhibitors under various conditions (Fig. 4). Furthermore, we demonstrated by cap-binding assays that the interaction between eIF4G and eIF4E, which initiates the eIF4F complex formation, was more resistant to GDC-0941 in 32D/ITD or STAT5A1*6-expressing 32D/TKD cells than in 32D/TKD or vector-control cells, respectively (Fig. 5B, C). These results strongly suggest that FLT3-ITD may sustain cap-dependent translation of *Mcl-1* mRNA in cells treated with the PI3K/Akt pathway inhibitors by protecting the eIF4E/eIF4G complex by maintaining 4EBP1 phosphorylation through robust STAT5 activation, although our data do not exclude the possibility that additional levels of Mcl-1 regulation contribute to the effects observed in this study. In this regard, it is interesting to note that dual PI3K/mTOR inhibitors, such as NVP-BEZ235, but not various PI3K inhibitors have been reported to downregulate Mcl-1 in various AML cells possibly through suppression of cap-dependent translation [32, 37].

The molecular mechanisms underlying the protective effect of STAT5 on the 4EBP1/eIF4F/Mcl-1 pathway have remained to be elucidated. As described above, the activation level of STAT5 neither correlated with Akt phosphorylation nor its sensitivity to the PI3K or Akt inhibitor (Fig. 4B, G), thus indicating that the protective effect should be mediated downstream of Akt. In this regard, it is interesting to note that FLT3-ITD has been reported to upregulate expression of Pim kinases, most likely through STAT5 [38, 39]. These serine/threonine kinases are implicated in upregulation of the mTORC1/4EBP1 pathway in hematopoietic cells through various mechanisms, including those involving phosphorylation of PRAS40, 4EBP1, and TSC2 [40–42]. Moreover, a recent study demonstrated that inhibition of Akt and Pim kinases synergistically inhibited mTORC1 signaling and Mcl-1 expression to induce apoptosis in various AML cells, although neither the synergistic effect nor expression levels of Pim kinases correlated with the presence of FLT3-ITD [43]. Further studies are in progress to address this and other possible mechanisms involved in protection of the eIF4E/eIF4G complex by FLT3-ITD through STAT5 in cells treated with the PI3K/Akt pathway inhibitors.

Various inhibitors for the PI3K/Akt pathway have shown only modest effects on AML cells, including those with FLT3-ITD, in preclinical and clinical studies, which may be partly explained by alternative mechanisms of activation of downstream effectors of the PI3K/Akt pathway [14]. The present study has revealed the sustained activation of 4EPB1/eIF4E/Mcl-1 pathway mediated by robust STAT5 activation as a mechanism for the resistance in AML cells with FLT3-ITD. To circumvent the relative resistance to the PI3K/Akt pathway inhibitors, co-administration of these inhibitors with chemotherapeutic agents or inhibitors directed against other signal transduction molecules has been evaluated in various studies [14]. For AML with FLT3-ITD, it has been reported that various FLT3 kinase inhibitors synergistically enhanced cytotoxic effects of the PI3K/Akt pathway inhibitors, including GDC-0941 and MK-2206 [44, 45]. It is noteworthy that these FLT3 inhibitors efficiently inhibited activation of STAT5, which may explain at least partly the synergistic effects reported. In the present study, we have demonstrated the synergistic enhancement of cytotoxic effects of the PI3K/Akt pathway inhibitors on AML cells with FLT3-ITD by selective inhibition of STAT5 by pimozide, which is widely used for treatment of neuropsychiatric disorders. Although the serum concentrations of pimozide in these patients are generally lower than 1 μM [28], the synergistic effects observed with low concentrations of pimozide and GDC-0941 (Fig. 2B) warrant future clinical studies to evaluate the efficacy of this strategy using pimozide. It is also noteworthy that other new STAT5 inhibitors including derivatives of pimozide with higher inhibitory effects are under preclinical or clinical studies [46–48] and that several PI3K/Akt pathway inhibitors including GDC-0941 and MK-2206 are currently under clinical studies [14–16].

Importantly, the combined effects of pimozide with GDC-0941 on the 4EBP1/Mcl-1 pathway as well as on viability was confirmed in primary AML cells with FLT3-ITD (Fig. 6). Although it could be evaluated in only two cases, both cases had relatively high allelic burdens (about 50%) of FLT3-ITD with their insertion sites in the TKD1 and exhibited very high peripheral blood count of blasts. Furthermore, we confirmed that the majority of FLT3 was activated constitutively with concomitant STAT5 activation, at least in the one case we examined (Fig. 6B). A recent report suggests that a very poor prognosis for AML with FLT3-ITD with this type of insertion could not be alleviated even by allogeneic hematopoietic stem cell transplantation [8]. Thus, the confirmation of the combined effects in the well-characterized cases with the dire prognosis further warrants future studies to develop the novel therapeutic strategy combining inhibitors targeting the PI3K/Akt pathway and STAT5.

## MATERIALS AND METHODS

### Cells and reagents

Ton.32D cells, a clone of murine IL-3-dependent 32Dcl3 cells transected with pTet-On (Clontech), and Ton.32D cells infected with pRevTRE-FLT3-D835Y, Ton.32D/FLT3-D835Y (32D/TKD), have been described previously [49] and were cultured in RPMI 1640 medium supplemented with 10% fetal calf serum (FCS) and 10% WEHI conditioned medium as the source of IL-3 or 5 U/ml recombinant mIL-3 (PeproTech, Rocky Hill, NJ). MV4–11 cells were purchased from ATCC and cultured in Iscove's modified Dulbecco medium (IMDM) containing 10% FCS.

The PI3K inhibitor GDC-0941 and the Akt inhibitor MK-2206 were purchased from Chemdea (Ridgewood, NJ) and Selleck (Houston, TX), respectively. ABT-737 was described previously [50]. The FLT3 inhibitor AC-220 and obatoclax were purchased from LC laboratories (Woburn, MA). DiOC6 was purchased from Invitrogen (Carlsbad, CA, USA). Etoposide was purchased from Wako (Tokyo, Japan). Doxycycline (DOX) and propidium iodide (PI) were purchased from Sigma Aldrich (St Louis, MO). The STAT5 inhibitor pimozide and antibodies against FLT3 (SC-479) and STAT5A (SC-1081) were purchased from Santa Cruz Biotechnology (Santa Cruz, CA). Monoclonal antibodies against phosphotyrosine (4G10) and ß-actin (A1978) were purchased from Upstate Biotechnology (Lake Placid, NY) and Sigma, respectively. An anti-Bax monoclonal antibody (YTH-6A7) was from Trevigen (Gaithersburg, MD, USA). Anti-Bcl-xL (610211) was purchased from BD Biosciences (San Jose, CA). Antibodies against cleaved Caspase-3 (CS-9661), Caspase-9 (CS-9508), cleaved Caspase-9 (CS-9509), phospho-Y694-STAT5 (CS-9359), Erk (CS-9102), phospho-T202/Y204-Erk (CS-9106), non-phospho-T46–4EBP1 (CS-4923), phospho-T37/46–4EBP1 (CS-2855), Akt (CS-4691), phospho-T308-Akt (CS-9275), phospho-S473-Akt (CS-9271), Mcl-1 (CS-5453), eIF4E (CS-2067), and eIF4G (CS-1469I were purchased from Cell Signaling (Beverly, MA).

### Expression plasmids, transfection, and infection

A retroviral plasmid for inducible expression of FLT3-ITD, pRevTRE-FLT3-ITD, was described previously [49]. A retroviral expression plasmid for a constitutively activated STAT5, pMX-puro-STAT5A1*6, and pMXs-puro were kindly provided by Dr. T. Kitamura [51]. A retroviral expression plasmid, pMXs-puro-Mcl-1, was constructed by subcloning the EcoRI/NotI fragment from pBS-Mcl-1 [52], a gift from Dr. S. Korsmeyer (Addgene plasmid #8763), into the EcoRI/NotI site of pMXs-puro.

Ton.32D/FLT3-ITD (32D/ITD) cells were obtained by infection of Ton.32D cells with pRevTRE-FLT3-ITD followed by selection in DOX-containing medium without IL-3, as described previously [49]. 32D/TKD cells expressing the constitutively activated STAT5 mutant STAT5A1*6 (32D/TKD/STATA1*6), overexpressing Mcl-1 (32D/TKD/Mcl-1), or vector control cells (32D/TKD/pMX) were obtained by infection of 32D/TKD cells with pMXs-puro-Mcl-1, pMX-puro-STAT5A1*6, or pMXs-puro, respectively, followed by selection with 1 μg/ml puromycin, as described previously [53]. These cells were cultured in medium containing DOX without IL-3 to proliferate dependent on FLT3-ITD or FLT3-TKD, but independent of mIL-3, to be analyzed in the following experiments.

### Analyses of cell proliferation and viability

Cell proliferation and viability were assessed by counting viable and nonviable cell numbers by the trypan blue-dye exclusion method. Cell viability was calculated by dividing number of viable cells by that of total cells. Viable cell numbers were also assessed by the sodium 3-[1-(phenylaminocarbonyl)-3, 4-tetrazolium]-bis (4-methoxy-6-nitro) benzene sulfonic acid hydrate (XTT) colorimetric assay using the Cell Proliferation Kit II (Roche Molecular Biochemicals, Mannheim, Germany), according to the manufacturer's instructions. For combination studies, the synergy was assessed with the combination index (CI) of Chou and Talalay method using Compu Syn software [30]. The CI value less than 0.9 indicate synergism. The potency of inhibitors was also analyzed by using Compu Syn software to obtain the median-effect dose (IC_50_) for each inhibitor.

### Flow cytometric analyses

For flow cytometric analysis of cell cycle and apoptosis, cells were treated with Krishan's reagent (0.05 mg/ml propidium iodide (PI), 0.1% sodium citrate, 0.02 mg/ml ribonuclease A, and 0.3% NP-40) for 30 min on ice and analyzed by flow cytometry.

Flow cytometric analyses of the Bax conformational change and Caspase-3 cleavage were performed using specific antibodies against activated Bax and cleaved Caspase-3 as described previously [50]. For flow cytometric analysis of Δψ_m_, cells were stained with DiOC6 and PI to be analyzed as described previously [50].

For flow cytometric analysis of non-phosphorylated 4EBP1, cells were fixed and permeabilized as described previously [50]. Cells were then incubated with anti non-phospho-T46–4EBP1 antibody on ice for 60 min, washed, and then incubated with R-phycoerythrin (PE)-conjugated goat F(ab’) anti-rabbit IgG(H+L) antibody (Southern Biotech, #4052–09) on ice for 30 min to be analyzed by flow cytometry. The data were analyzed by the FlowJo software (Tree Star. Inc., Ashland, OR).

### Immunoblotting and cap-binding assays

For immunoblotting experiments, cells were lysed in a lysis buffer containing 1% Triton X-100, 20 mM Tris-HCl (pH 7.5), 150 mM NaCl, 1 mM EDTA, 1 mM sodium orthovanadate, 1 mM phenylmethylsulfonyl fluoride and 10 μg/ml each of aprotinin and leupeptin at 4°C for 15 min. Cell lysates were subjected to immunoblot analysis essentially as described previously [54]. For cap-binding experiments, cell lysates were incubated overnight at 4ºC with 7-methyl-GTP-sepharose beads (Jena Bioscience, Jena, Germany). The beads were washed extensively with the lysis buffer and heated at 100ºC for 5 min in 1 X Laemmli's buffer, and eluted proteins were subjected to immunoblot analysis.

### Analyses of primary AML cells

Mononuclear cells were isolated by centrifugation through Ficoll-Hypaque from the peripheral blood derived from FLT3-ITD-positive AML patients at diagnosis with white blood cell count of 149, 000/μl with 80% blasts or 94, 800/μl with 81% blasts (Case 1 or 2, respectively). Cryopreserved cells were thawed and cultured for 1 day in IMDM with 10% FCS before analyses. The ITD mutation was detected by the RT-PCR method described previously [55] and was further analyzed by sequencing cloned RT-PCR products.

The study was approved by the ethical committee of Tokyo Medical and Dental University. Written informed consent was obtained from the patient in compliance with the Declaration of Helsinki.

## SUPPLEMENTARY FIGURES



## References

[1] Stirewalt DL, Radich JP (2003). The role of FLT in haematopoietic malignancies. Nature reviews Cancer.

[2] Meshinchi S, Appelbaum FR (2009). Structural and functional alterations of FLT3 in acute myeloid leukemia. Clin Cancer Res.

[3] Kindler T, Lipka DB, Fischer T (2010). FLT3 as a therapeutic target in AML: still challenging after all these years. Blood.

[4] Sudhindra A, Smith CC (2014). FLT3 inhibitors in AML: are we there yet?. Current hematologic malignancy reports.

[5] Kayser S, Schlenk RF, Londono MC, Breitenbuecher F, Wittke K, Du J, Groner S, Spath D, Krauter J, Ganser A, Dohner H, Fischer T, Dohner K (2009). German-Austrian AMLSG. Insertion of FLT3 internal tandem duplication in the tyrosine kinase domain-1 is associated with resistance to chemotherapy and inferior outcome. Blood.

[6] Schnittger S, Bacher U, Haferlach C, Alpermann T, Kern W, Haferlach T (2012). Diversity of the juxtamembrane and TKD1 mutations (exons 13–15) in the FLT3 gene with regards to mutant load, sequence, length, localization, and correlation with biological data. Genes, chromosomes & cancer.

[7] Pratcorona M, Brunet S, Nomdedeu J, Ribera JM, Tormo M, Duarte R, Escoda L, Guardia R, Queipo de Llano MP, Salamero O, Bargay J, Pedro C, Marti JM (2013). Favorable outcome of patients with acute myeloid leukemia harboring a low-allelic burden FLT3-ITD mutation and concomitant NPM1 mutation: relevance to post-remission therapy. Blood.

[8] Schlenk RF, Kayser S, Bullinger L, Kobbe G, Casper J, Ringhoffer M, Held G, Brossart P, Lubbert M, Salih HR, Kindler T, Horst HA, Wulf G (2014). Differential impact of allelic ratio and insertion site in FLT3-ITD positive AML with respect to allogeneic hematopoietic stem cell transplantation. Blood.

[9] Hayakawa F, Towatari M, Kiyoi H, Tanimoto M, Kitamura T, Saito H, Naoe T (2000). Tandem-duplicated Flt3 constitutively activates STAT5 and MAP kinase and introduces autonomous cell growth in IL-3-dependent cell lines. Oncogene.

[10] Mizuki M, Fenski R, Halfter H, Matsumura I, Schmidt R, Müller C, Grüning W, Kratz-Albers K, Serve S, Steur C, Büchner T, Kienast J, Kanakura Y (2000). Flt3 mutations from patients with acute myeloid leukemia induce transformation of 32D cells mediated by the Ras and STAT5 pathways. Blood.

[11] Choudhary C, Schwäble J, Brandts C, Tickenbrock L, Sargin B, Kindler T, Fischer T, Berdel WE, Müller-Tidow C, Serve H (2005). AML-associated Flt3 kinase domain mutations show signal transduction differences compared with Flt3 ITD mutations. Blood.

[12] Janke H, Pastore F, Schumacher D, Herold T, Hopfner KP, Schneider S, Berdel WE, Büchner T, Woermann BJ, Subklewe M, Bohlander SK, Hiddemann W, Spiekermann K, Polzer H (2014). Activating FLT3 mutants show distinct gain-of-function phenotypes *in vitro* and a characteristic signaling pathway profile associated with prognosis in acute myeloid leukemia. PLoS One.

[13] Martelli AM, Evangelisti C, Chiarini F, McCubrey JA (2010). The phosphatidylinositol 3-kinase/Akt/mTOR signaling network as a therapeutic target in acute myelogenous leukemia patients. Oncotarget.

[14] Polak R, Buitenhuis M (2012). The PI3K/PKB signaling module as key regulator of hematopoiesis: implications for therapeutic strategies in leukemia. Blood.

[15] Yap TA, Yan L, Patnaik A, Fearen I, Olmos D, Papadopoulos K, Baird RD, Delgado L, Taylor A, Lupinacci L, Riisnaes R, Pope LL, Heaton SP (2011). First-in-man clinical trial of the oral pan-AKT inhibitor MK-2206 in patients with advanced solid tumors. Journal of clinical oncology: official journal of the American Society of Clinical Oncology.

[16] Sarker D, Ang JE, Baird RD, Kristeleit R, Shah K, Moreno Garcia V, Clarke PA, Raynaud FI, Levy G, Ware JA, Mazina K, Lin R, Wu J (2014). First-in-human Phase I study of Pictilisib (GDC-0941), a potent pan-class I phosphatidylinositol-3-kinase (PI3K) inhibitor, in patients with advanced solid tumors. Clin Cancer Res.

[17] Altman JK, Sassano A, Platanias LC (2011). Targeting mTOR for the treatment of AML. New agents and new directions. Oncotarget.

[18] Laplante M, Sabatini DM (2012). mTOR signaling in growth control and disease. Cell.

[19] Chen W, Drakos E, Grammatikakis I, Schlette EJ, Li J, Leventaki V, Staikou-Drakopoulou E, Patsouris E, Panayiotidis P, Medeiros LJ, Rassidakis GZ (2010). mTOR signaling is activated by FLT3 kinase and promotes survival of FLT3-mutated acute myeloid leukemia cells. Mol Cancer.

[20] Mills JR, Hippo Y, Robert F, Chen SM, Malina A, Lin CJ, Trojahn U, Wendel HG, Charest A, Bronson RT, Kogan SC, Nadon R, Housman DE (2008). mTORC1 promotes survival through translational control of Mcl-1. Proc Natl Acad Sci U S A.

[21] Hsieh AC, Costa M, Zollo O, Davis C, Feldman ME, Testa JR, Meyuhas O, Shokat KM, Ruggero D (2010). Genetic dissection of the oncogenic mTOR pathway reveals druggable addiction to translational control via 4EBP-eIF4E. Cancer Cell.

[22] Martelli AM, Evangelisti C, Chappell W, Abrams SL, Basecke J, Stivala F, Donia M, Fagone P, Nicoletti F, Libra M, Ruvolo V, Ruvolo P, Kempf CR (2011). Targeting the translational apparatus to improve leukemia therapy: roles of the PI3K/PTEN/Akt/mTOR pathway. Leukemia.

[23] Glaser SP, Lee EF, Trounson E, Bouillet P, Wei A, Fairlie WD, Izon DJ, Zuber J, Rappaport AR, Herold MJ, Alexander WS, Lowe SW, Robb L, Strasser A (2012). Anti-apoptotic Mcl-1 is essential for the development and sustained growth of acute myeloid leukemia. Genes & development.

[24] Gores GJ, Kaufmann SH (2012). Selectively targeting Mcl-1 for the treatment of acute myelogenous leukemia and solid tumors. Genes & development.

[25] Yoshimoto G, Miyamoto T, Jabbarzadeh-Tabrizi S, Iino T, Rocnik JL, Kikushige Y, Mori Y, Shima T, Iwasaki H, Takenaka K, Nagafuji K, Mizuno S, Niiro H (2009). FLT3-ITD up-regulates MCL-1 to promote survival of stem cells in acute myeloid leukemia via FLT3-ITD-specific STAT5 activation. Blood.

[26] Kasper S, Breitenbuecher F, Heidel F, Hoffarth S, Markova B, Schuler M, Fischer T (2012). Targeting MCL-1 sensitizes FLT3-ITD-positive leukemias to cytotoxic therapies. Blood cancer journal.

[27] Spiekermann K, Bagrintseva K, Schwab R, Schmieja K, Hiddemann W (2003). Overexpression and constitutive activation of FLT3 induces STAT5 activation in primary acute myeloid leukemia blast cells. Clin Cancer Res.

[28] Nelson EA, Walker SR, Weisberg E, Bar-Natan M, Barrett R, Gashin LB, Terrell S, Klitgaard JL, Santo L, Addorio MR, Ebert BL, Griffin JD, Frank DA (2011). The STAT5 inhibitor pimozide decreases survival of chronic myelogenous leukemia cells resistant to kinase inhibitors. Blood.

[29] Nelson EA, Walker SR, Xiang M, Weisberg E, Bar-Natan M, Barrett R, Liu S, Kharbanda S, Christie AL, Nicolais M, Griffin JD, Stone RM, Kung AL, Frank DA (2012). The STAT5 Inhibitor Pimozide Displays Efficacy in Models of Acute Myelogenous Leukemia Driven by FLT3 Mutations. Genes & cancer.

[30] Chou TC, Talalay P (1984). Quantitative analysis of dose-effect relationships: the combined effects of multiple drugs or enzyme inhibitors. Adv Enzyme Regul.

[31] Thomas LW, Lam C, Edwards SW (2010). Mcl-1, the molecular regulation of protein function. FEBS letters.

[32] Thomas D, Powell JA, Vergez F, Segal DH, Nguyen NY, Baker A, Teh TC, Barry EF, Sarry JE, Lee EM, Nero TL, Jabbour AM, Pomilio G (2013). Targeting acute myeloid leukemia by dual inhibition of PI3K signaling and Cdk9-mediated Mcl-1 transcription. Blood.

[33] Nguyen M, Marcellus RC, Roulston A, Watson M, Serfass L, Murthy Madiraju SR, Goulet D, Viallet J, Belec L, Billot X, Acoca S, Purisima E, Wiegmans A (2007). Small molecule obatoclax (GX15–070) antagonizes MCL-1 and overcomes MCL-1-mediated resistance to apoptosis. Proc Natl Acad Sci U S A.

[34] van Delft MF, Wei AH, Mason KD, Vandenberg CJ, Chen L, Czabotar PE, Willis SN, Scott CL, Day CL, Cory S, Adams JM, Roberts AW, Huang DC (2006). The BH3 mimetic ABT-737 targets selective Bcl-2 proteins and efficiently induces apoptosis via Bak/Bax if Mcl-1 is neutralized. Cancer Cell.

[35] Ferbeyre G, Moriggl R (2011). The role of Stat5 transcription factors as tumor suppressors or oncogenes. Biochimica et biophysica acta.

[36] Wang JM, Chao JR, Chen W, Kuo ML, Yen JJ, Yang-Yen HF (1999). The antiapoptotic gene mcl-1 is up-regulated by the phosphatidylinositol 3-kinase/Akt signaling pathway through a transcription factor complex containing CREB. Mol Cell Biol.

[37] Chapuis N, Tamburini J, Green AS, Vignon C, Bardet V, Neyret A, Pannetier M, Willems L, Park S, Macone A, Maira SM, Ifrah N, Dreyfus F (2010). Dual inhibition of PI3K and mTORC1/2 signaling by NVP-BEZ235 as a new therapeutic strategy for acute myeloid leukemia. Clin Cancer Res.

[38] Mizuki M, Schwable J, Steur C, Choudhary C, Agrawal S, Sargin B, Steffen B, Matsumura I, Kanakura Y, Bohmer FD, Muller-Tidow C, Berdel WE, Serve H (2003). Suppression of myeloid transcription factors and induction of STAT response genes by AML-specific Flt3 mutations. Blood.

[39] Kim KT, Baird K, Ahn JY, Meltzer P, Lilly M, Levis M, Small D (2005). Pim-1 is up-regulated by constitutively activated FLT3 and plays a role in FLT3-mediated cell survival. Blood.

[40] Tamburini J, Green AS, Bardet V, Chapuis N, Park S, Willems L, Uzunov M, Ifrah N, Dreyfus F, Lacombe C, Mayeux P, Bouscary D (2009). Protein synthesis is resistant to rapamycin and constitutes a promising therapeutic target in acute myeloid leukemia. Blood.

[41] Zhang F, Beharry ZM, Harris TE, Lilly MB, Smith CD, Mahajan S, Kraft AS (2009). PIM1 protein kinase regulates PRAS40 phosphorylation and mTOR activity in FDCP1 cells. Cancer biology & therapy.

[42] Lu J, Zavorotinskaya T, Dai Y, Niu XH, Castillo J, Sim J, Yu J, Wang Y, Langowski JL, Holash J, Shannon K, Garcia PD (2013). Pim2 is required for maintaining multiple myeloma cell growth through modulating TSC2 phosphorylation. Blood.

[43] Meja K, Stengel C, Sellar R, Huszar D, Davies BR, Gale RE, Linch DC, Khwaja A (2014). PIM and AKT kinase inhibitors show synergistic cytotoxicity in acute myeloid leukaemia that is associated with convergence on mTOR and MCL1 pathways. British journal of haematology.

[44] Jin L, Tabe Y, Lu H, Borthakur G, Miida T, Kantarjian H, Andreeff M, Konopleva M (2013). Mechanisms of apoptosis induction by simultaneous inhibition of PI3K and FLT3-ITD in AML cells in the hypoxic bone marrow microenvironment. Cancer letters.

[45] Weisberg E, Liu Q, Zhang X, Nelson E, Sattler M, Liu F, Nicolais M, Zhang J, Mitsiades C, Smith RW, Stone R, Galinsky I, Nonami A (2013). Selective Akt inhibitors synergize with tyrosine kinase inhibitors and effectively override stroma-associated cytoprotection of mutant FLT3-positive AML cells. PLoS One.

[46] Hayakawa F, Sugimoto K, Harada Y, Hashimoto N, Ohi N, Kurahashi S, Naoe T (2013). A novel STAT inhibitor, OPB-31121, has a significant antitumor effect on leukemia with STAT-addictive oncokinases. Blood cancer journal.

[47] Pinz S, Unser S, Rascle A (2014). The natural chemopreventive agent sulforaphane inhibits STAT5 activity. PLoS One.

[48] Rondanin R, Simoni D, Romagnoli R, Baruchello R, Marchetti P, Costantini C, Fochi S, Padroni G, Grimaudo S, Pipitone RM, Meli M, Tolomeo M (2014). Inhibition of activated STAT5 in Bcr/Abl expressing leukemia cells with new pimozide derivatives. Bioorganic & medicinal chemistry letters.

[49] Oshikawa G, Nagao T, Wu N, Kurosu T, Miura O (2011). c-Cbl and Cbl-b ligases mediate 17-allylaminodemethoxygeldanamycin-induced degradation of autophosphorylated Flt3 kinase with internal tandem duplication through the ubiquitin proteasome pathway. J Biol Chem.

[50] Kurosu T, Ohki M, Wu N, Kagechika H, Miura O (2009). Sorafenib induces apoptosis specifically in cells expressing BCR/ABL by inhibiting its kinase activity to activate the intrinsic mitochondrial pathway. Cancer Res.

[51] Onishi M, Nosaka T, Misawa K, Mui AL, Gorman D, McMahon M, Miyajima A, Kitamura T (1998). Identification and characterization of a constitutively active STAT5 mutant that promotes cell proliferation. Mol Cell Biol.

[52] Rinkenberger JL, Horning S, Klocke B, Roth K, Korsmeyer SJ (2000). Mcl-1 deficiency results in peri-implantation embryonic lethality. Genes & development.

[53] Nagao T, Oshikawa G, Wu N, Kurosu T, Miura O (2011). DNA damage stress and inhibition of Jak2-V17F cause its degradation and synergistically induce apoptosis through activation of GSK3beta. PLoS One.

[54] Miura O, Cleveland JL, Ihle JN (1993). Inactivation of erythropoietin receptor function by point mutations in a region having homology with other cytokine receptors. Mol Cell Biol.

[55] Kiyoi H, Naoe T, Nakano Y, Yokota S, Minami S, Miyawaki S, Asou N, Kuriyama K, Jinnai I, Shimazaki C, Akiyama H, Saito K, Oh H (1999). Prognostic implication of FLT3 and N-RAS gene mutations in acute myeloid leukemia. Blood.

