# Supplementary material for: FLT3-ITD confers resistance to the PI3K/Akt pathway inhibitors by protecting the mTOR/4EBP1/Mcl-1 pathway through STAT5 activation in acute myeloid leukemia
Source: Oncotarget. 2015 Mar 16;6(11):9189–205. doi: 10.18632/oncotarget.3279 (PMC4496211; doi:10.18632/oncotarget.3279)
Supplement: Supplementary file 1 [file oncotarget-06-9189-s001.pdf]

SUPPLEMENTARY FIGURES

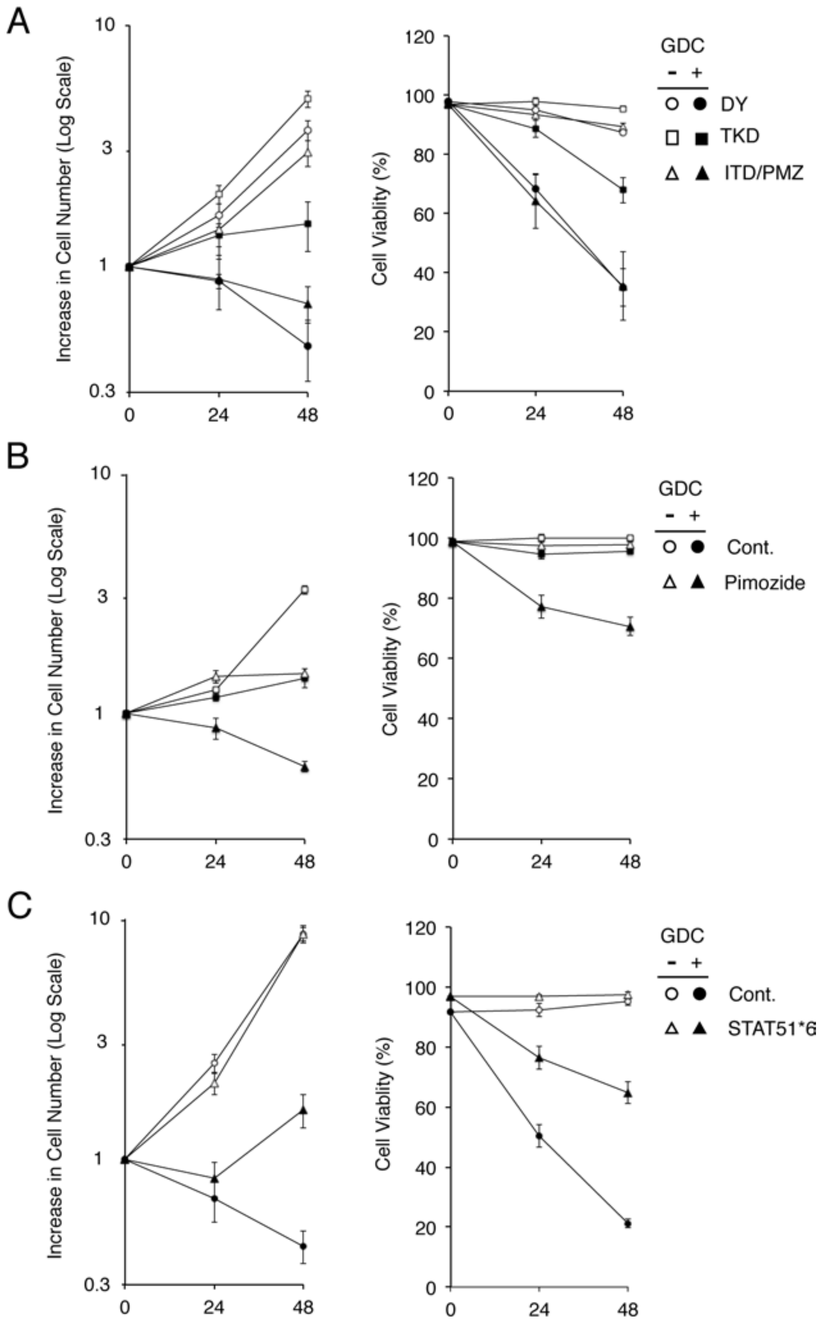

Supplementary Figure S1: FLT3-ITD confers resistance to GDC-0941 for inhibition of cell proliferation and survival, which is abolished by pimozide and conferred also on FLT3-TKD-expressing cells by STAT5A1*6. A. 32D/ITD (ITD) or 32D/TKD (TKD) cells were plated at 1 X 10^5/ml with or without 2 µM GDC-0941 (GDC) or 3 µM pimozide (PMZ), as indicated. After cultured for indicated times, viable cell number and viability were counted and plotted. Each data point represents the mean of triplicate determinations, with error bars indicating standard errors. **B.** MV4-11 cells were culture with or without 1µM GDC-0941 (GDC) or 3 µM pimozide, as indicated, and analyzed. **C.** 32D/TKD cells transduced with STAT5A1*6 (STAT51*6) or vector control cells, as indicated, were cultured with or without 2µM GDC-0941, as indicated, and analyzed.

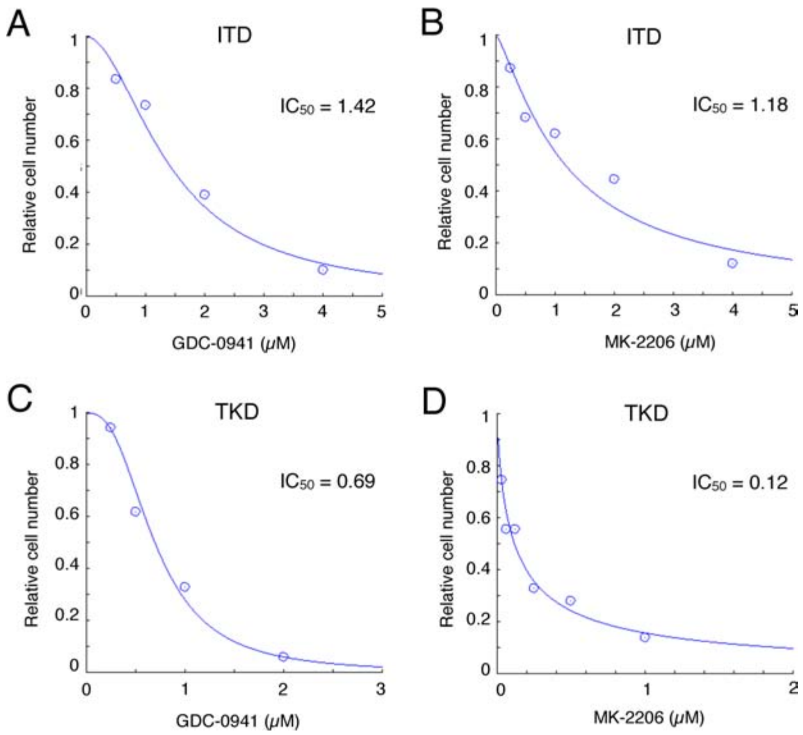

Supplementary Figure S2: 32D/ITD cells are more resistant than 32D/TKD cells to GDC-0941 and MK-2206 for inhibition of cell proliferation. 32D/ITD (ITD) cells in **A, B.** or 32D/TKD (TKD) cells in **C, D.** were cultured with indicated concentrations of GDC-0941 **(A, C)** or MK-2206 **(B, D)** for 48 h. Viable cell numbers were measured by the XTT colorimetric assay. Each column represents the mean of triplicate determinations, with the dose-effect curves and the median-effect dose (IC_{50}) obtained by Compu Syn Software.
